# Effectiveness and process evaluation in obesity and type 2 diabetes prevention programs in children: a systematic review and meta-analysis

**DOI:** 10.1186/s12889-021-10297-8

**Published:** 2021-02-12

**Authors:** M. Seral-Cortes, P. De Miguel-Etayo, P. Zapata, M. L. Miguel-Berges, L. A. Moreno

**Affiliations:** 1 Growth, Exercise, NUtrition and Development (GENUD) Research Group, Instituto Agroalimentario de Aragón (IA2), Instituto de Investigación Sanitaria, Aragón (IIS Aragón), Universidad de Zaragoza, 50009 Zaragoza, Spain; 2 Department of Psychiatry and Nursing, Faculty of Health Sciences, Universidad de Zaragoza, Zaragoza, Spain; 3 CIBER Fisiopatología de la Obesidad y Nutrición (CIBERobn), Instituto de Salud Carlos III, Madrid, Spain; 4 Departamento de nutrición y dietética, Universidad de Antioquia, Medellín, Colombia

**Keywords:** Process evaluation, Obesity, Type 2 diabetes mellitus, Body composition, Health plan implementation, Primary prevention

## Abstract

**Background:**

Obesity in children is one of the most severe public health challenges of the current century and Type 2 Diabetes Mellitus (T2DM) frequency is also escalating. More so, the importance of process evaluation (PE) in complex interventions is increasingly recognized. The present review, aims to identify the effectiveness in terms of body composition parameters in a generation of articles to prevent obesity and T2DM in children. We hypothesise that those studies reporting PE applying the latest implementation guidelines suggested by the researchers would potentially show positive changes in body composition compared to those not reporting it. Additionally, we will evaluate the implementation degree of PE in those articles considering it and describe the PE subcomponents. Lastly, we aim to assess the intervention target used and its results.

**Methods:**

A literature review was performed in parallel by 2 independent reviewers. A final number of 41 studies were selected for inclusion criteria.

**Results:**

Meta-analysis of BMI and zBMI found non-significant effects of the proposed interventions. Sub-group analysis revealed only a significant effect in studies which performed PE. Moreover, PE was reported in 42% effective studies and 57% non-effective studies. Fidelity and satisfaction were the most implemented PE subcomponents, although there was a generally low grade of PE use (7/41). The highest proportion of effectiveness (83%) was shown in interventions of physical activity alone while the intervention most used was 3-arm target (diet, PA and BS).

**Conclusions:**

Overall, obesity and T2DM prevention studies included in this review are not effective in terms of BMI and zBMI. Those studies performing PE reported to be effective in terms of BMI, while studies not reporting PE did not have positive results in terms of BMI and zBMI. In addition, none of the intervention studies included all PE indicators and most studies, which included PE in their interventions, did not provide full report of the PE components, according to the guidelines used for the present review. PROSPERO registration number: CRD42018093667.

**Supplementary Information:**

The online version contains supplementary material available at 10.1186/s12889-021-10297-8.

## Background

Obesity has important effects on health in the short, medium and long terms where metabolic complications are common [1]. In children, overweight and obesity’s prevalence has increased in every continent of the world in recent decades [2]. Currently over 1.5 billion adults and 170 million children are overweight or obese [3]. Obesity in children is one of the most severe global public health challenges of the current century [4]. Type 2 diabetes mellitus (T2DM) frequency is rapidly escalating, with vital implications for morbidity and mortality. Worldwide prevalence figures of T2DM in 2019 estimates 463 million (9.3%) people affected, escalating to 578 million (10.2%) by 2030 and 700 million (10.9%) by 2045 [5]. As a result of the increasing rates of obesity and sedentary lifestyle, younger populations are promptly diagnosed with T2DM [6] corresponding to the extensive spread of childhood obesity [7].

Lifestyle intervention programs for the treatment of children and adolescents with obesity had a long-term success rate lower than 10% [8]. The inefficiency of prevention programs of lifestyle intervention comprehends several factors including lack of multidisciplinary approaches, increasing mental health related issues or insufficient involvement of the parents, which might interact with the program’s adherence [9]. As T2DM is one of the most frequent metabolic complications of obesity with important long-term effects, combined actions to prevent both obesity and T2DM could be developed.

A number of research activities aim to build and evaluate evidence-based programs to prevent childhood obesity [10]. The school is usually regarded as a suitable and effective setting to carry out obesity prevention programs aiming to evaluate students’ energy balance-related behaviors (EBRBs) [11]. However, most of systematic reviews conducted in United states and Europe of school-based interventions preventing obesity, promote physical activity (PA) and decrease sedentary behaviors, show moderate evidence of effectiveness [12, 13]. Most of the obesity prevention programs are difficult to implement and evaluate due to the multiple interacting components that exist. Randomized controlled trials (RCT) of these programs are often known for its difficulties to find out the reason why the program worked or did not work without examining underlying processes [11]. With public health depending on the impact of these programs and their implementation in practice, it is essential to interpret whether a program was implemented as intended, to what extend and how these concepts could modulate the effectiveness of the program [14]. Verifying that the interventions are delivered as planned as well as factors affecting implementation, allows an accurate interpretation of intervention outcomes and context by the researchers and policy makers in order to optimize further implementation of interventions in the future [15].

The importance of process evaluation (PE) in public health intervention research is gradually recognized [16]. The use of PE is to observe and record program implementation as well as helping to understand the connection between specific program elements and program outcomes. Several practical frameworks and models are available to lead different professionals to the development of an evaluation plan with wide scope, including PE. There are comprehensive and systematic approaches for developing a PE plan to assess the implementation of a particular prevention program intervention. These approaches are divided in different indicators including recruitment, reach, fidelity, dose and satisfaction [16]. However, there is no agreement on what is the ideal standard to classify the study of implementation into key parts, for instance dose and reach, and it is not possible to produce a definite standard among the various frameworks used at present. Without the presence of PE, it is challenging to differentiate between outcomes that are, in theory, related to a lack of fidelity and those ones attributed to the incompetence of the intervention to achieve the expected results. Unsuccessful attempts to perform an intervention as intended prompts to misleading results and conclusions about the effectiveness of the intervention and is considered not valid to replicate to use in future investigations in the scientific community. Multicomponent prevention programs are complex interventions, designed to work synergistically. For this reason, PE contributes to interpret complex outcome effects and helps to understand the analysis of the intervention delivered [17, 18].

RCTs are considered the best study design to establish the effectiveness of interventions with certain degree of complexity. However, there is no information at present of how an intervention might be reproduced in a particular context, or whether trial outcomes will be replicated. To our knowledge, there is no systematic review and meta-analysis focusing on the evaluation of effectiveness and the development and report of PE of interventions in health programs preventing obesity and T2DM in children. The present review, aims to identify the effectiveness in terms of body composition parameters in a generation of articles to prevent obesity and T2DM in children. We hypothesise that those studies reporting PE applying the latest implementation guidelines suggested by the researchers [19, 20] would potentially be more effective in terms of changes in body composition compared to those not reporting it. Additionally, we will evaluate the implementation degree of PE in those articles considering it and describe the PE subcomponents. Lastly, we aim to assess the intervention target used and its results.

## Methods

### Literature search

A literature review was performed in parallel by 2 independent reviewers and a third independent reviewer was involved when inconsistency or disagreement with the selection of articles was identified. The protocol was developed according to the preferred reporting items for systematic reviews and meta-analysis (PRISMA) guidelines adapted to the design of the present study [21]. Moreover, the systematic literature search was registered on the International Prospective Register of Systematic Reviews (PROSPERO, registration number: CRD42018093667). An in-depth search of electronic databases was conducted in the PubMed, Scopus and Embase. (Mesh®) terms were used during the search strategy in PUBMED, based on medical subject headings and text words of peer papers identified. Search terms and text words are described in full report as follows: ((((((“Diabetes Mellitus, Type 2”[Mesh]) OR “Risk”[Mesh]) OR (“Obesity”[Mesh] OR “Pediatric Obesity”[Mesh] OR “Obesity, Abdominal”[Mesh] OR “Obesity Management”[Mesh])) AND “Health Plan Implementation”[Mesh]) OR (“prevention and control” [Subheading] OR “Primary Prevention”[Mesh])) OR (“Outcome and Process Assessment (Health Care)”[Mesh] OR “Process Assessment (Health Care)”[Mesh])) AND “Body Composition”[Mesh])))))). The reference lists of all included papers were doublechecked to identify potential missing articles that might have been missed during the initial search. The focus was on the studies assessing the effectiveness on changes in body composition: zBMI (body mass index z score) and/or BMI (body mass index) and/or waist circumference as well as the consideration of any of the PE subcomponents in the health program intervention.

Other risk factors such as high and increased blood pressure, high and increased blood glucose level, insulin level, fat-free mass, percentage of android mass and percentage obesity fat were also considered in the selection of articles as secondary outcomes. Articles were also considered if any of the primary outcomes were referred as secondary outcome and vice versa.

### Data extraction and quality assessment

**Fig. 1:**
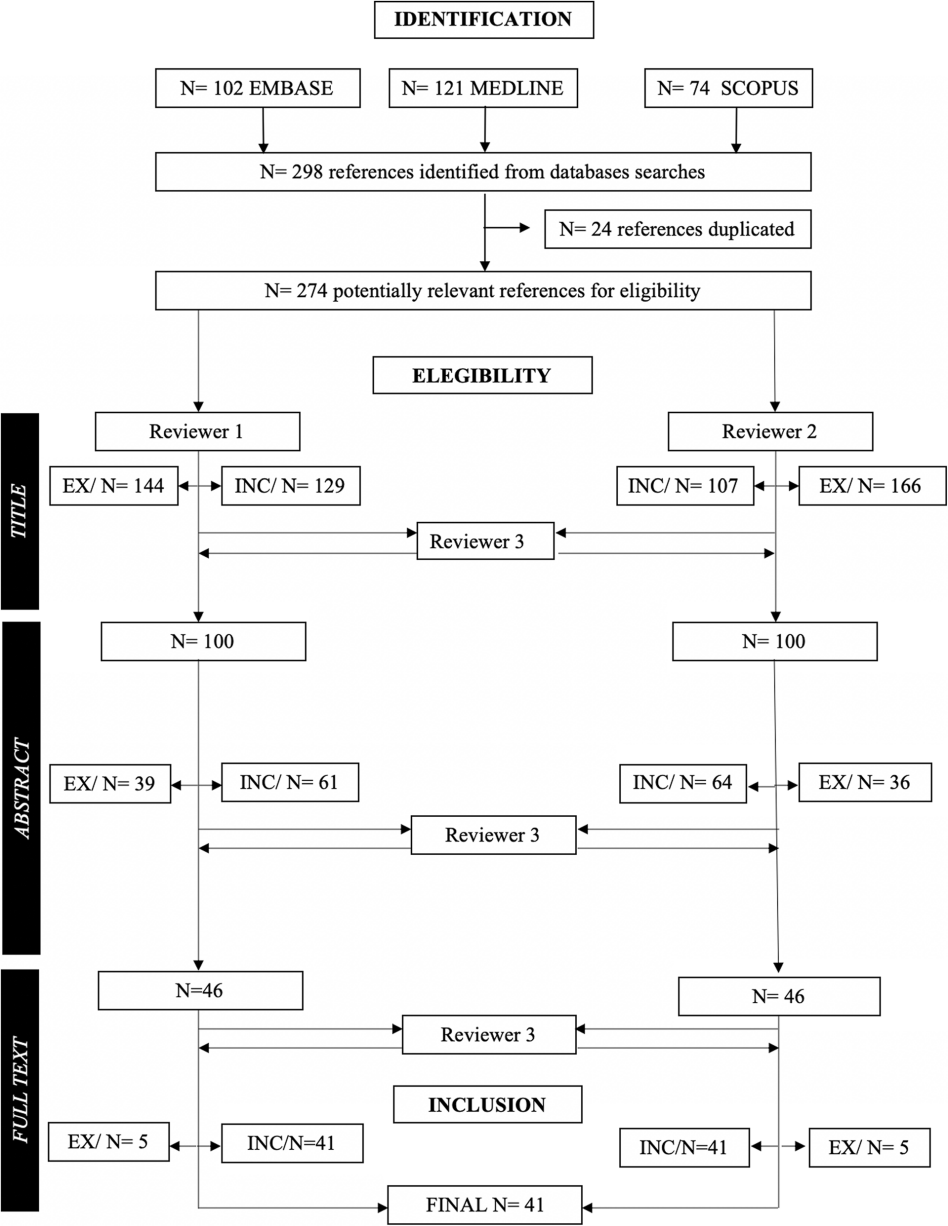
PRISMA flow chart of search strategy process

The systematic selection process was performed in 3 phases (Fig. 1). Final results are presented in the description of papers. The inclusion criteria were 1) presence of obesity and/or T2DM parameters as primary or secondary outcomes, 2) diet, physical activity (PA) and behavioral support (BS) alone or combined with other kind of intervention, 3) population children age 6–12 years old, 4) written in English, 5) published from 2008 and 6) exclusively randomized control trials. Any discrepancies with the inclusion criteria between reviewers was discussed to reach a common final consensus.

As far as exclusion criteria is concerned it was excluded from the review 1) clinical populations (disorders, disabled, institutionalized) or presence of Type 1 diabetes mellitus and other types of diabetes (E.g. insulin-dependent-diabetes, pregnancy diabetes, gestational diabetes), 2) no intervention applied, 3) children younger than 6 years and older populations than 12 years old, 4) not wrote in English language, 5) published before 2008 and 6) non randomized control trials and unpublished studies.

Studies were classified depending on an established process according to the QUALYST (Standard Quality Assessment Criteria for evaluating primary research papers from a variety of fields) checklist for measuring quality by 2 independent reviewers. The mentioned checklist has 14 questions which have to be answered with “yes”, “partial”, “no” or “not applicable” depending on the quality of each article. The summary score is the total of the accumulated answers transcribed into a number between 0 and 1 indicating the quality of each publication, being 1 the highest possible result. When comparting the methodological score between the 2 reviewers, a third reviewer intervened in the event of a numerical difference of more than 0.2 within the same publication evaluated.

The Cochrane Collaborations Tool for assessing risk of bias in randomized trials [22] and the Cochrane Handbook for Systematic Reviews of Interventions [23] were used to assess methodological risk of bias for randomized control trials, which recommend the explicit reporting of the following domains: random sequence generation, allocation sequence concealment, blinding (participants and personnel), blinding (outcome assessment), completeness of outcome data, selective reporting and other sources of bias. Each item was given a score as being at high, low or unclear risk of bias as per criteria provided [23].

### Meta-analysis

Within included reviews, a meta-analysis of 30 studies reporting BMI and 16 studies reporting zBMI in an intervention population versus a comparator population were undertaken. Meta-analysis of subgroups according to PE reporting was performed in order to identify disparities in studies between the 2 groups including PE or not and the report of its effectiveness. Within included studies for meta-analysis, Greening et al. [24] and Kalavainen et al. [25] were not included on BMI and Lison et al. [26] was not considered as not meeting the inclusion criteria for BMI and zBMI.

Standardized mean difference was the appropriate metric for the data type. The interventions compared in meta-analysis were changes in BMI and zBMI in the intervention group versus changes in BMI and zBMI in the control group. Sub-group analysis were carried out in each outcome studying PE as factor covariate to observe potential differences between the groups implementing PE versus those not implementing it.

Data on mean difference in BMI and zBMI between intervention and comparator groups and standard deviation of the difference from studies that reported data in a comparable way were analyzed in OpenMetaAnalyst software using inverse variance random-effects meta-analysis. Continuous random-effects DerSimonian-Laird analysis were selected to reflect different study groups, setting, and age among the included studies.

The confidence level used was 95.0. I2 statistic was used to assess the heterogeneity of the studies [23]. This statistic explains the variance within studies as a proportion of the total variance. < 25% value showed low heterogeneity, 25 to 50% value showed moderate heterogeneity, > 50 to 75% value showed high heterogeneity and > 75% value indicated very high heterogeneity. Associated *p*-values were also displayed, and significance level was set at *p* < 0.05, showing heterogeneity when *p*-values were below 0.05.

### Process evaluation indicators and criteria

The present systematic review has evaluated the PE implementation based on the guidelines provided by Saunders et al. [19] and Moore et al. [20]. In short, both guidelines share the relevance of assessing fidelity, dose and reach indicators. Saunders et al. provides a more detailed list of indicators and its use, considering recruitment and context and 2 dose categories (delivered and received). Moore et al. described a framework of PE built on 3 themes described in the 2008 MRC guidance (implementation, mechanisms and context) [27]. When applying the criteria of these guidelines in our articles, we observed that fidelity was considered mainly from the caregivers feedback to assess the extent of the intervention implementation according to the initial study program; moreover, dose was reported to see the mode of the program delivery in terms of training, intervention components, materials and content through control sessions from the staff (delivered), and also in terms of use and reaction of children and parents to the activities delivered through questionnaires (received). Finally, reach evaluated the participants attendance and to assess the program’s effect on the targeted group, also through questionnaires. However, there might be different effects depending on which context the intervention is performed.

## Results

### Description of papers

The selection process is displayed in Fig. 1. In summary, the screening process was divided in 3 stages: identification, eligibility and inclusion. First, after deleting 24 duplicates, identification stage left 273 articles for inclusion. After title and abstract screening, 46 articles were included. Finally, the inclusion stage showed, after full text reading, a final 41 (15%) articles [24–26, 28–65] which main characteristics are summarized in Table 1. From the selected articles, 39 of them focused on obesity and 2 articles on T2DM, although 7 articles from obesity included glucose and insulin levels in their main research parameters.

**Table 1:** Main characteristics of the included studies

Author/Year	Country	Sample Size	Gender	Mean Age	Setting	Intervention duration
Croker et al. 2012 [28]	UK	72	Not reported	10.3 ± 1.6	Hospital	12 months
Danielsen et al. 2011 [29]	Norway	49	24 male; 25 female	10.6 ± 1.2	Family based/primary care	12 months
Davis et al. 2012 [30]	USA	222	93 male; 129 female	9.4 ± 1.1	School	10–15 weeks
Eather et al. 2013 [31]	Australia	213	108 male; 105 female	10.7 ± 0.6	School	8 weeks
Elder et al. 2013 [32]	UK	541	243 male; 298 female	6.6 ± 0.7	Recreation Center	2 years
Foley et al. 2016 [33]	New Zealand	251	142 male; 109 female	11.25	Family based/Home	6 months
Gerards et al. 2015 [34]	Netherlands	86	38 male; 48 female	7.2 ± 1.4	School	14 weeks
Hollis et al. 2016 [35]	Australia	1150	male 543; female 583	12.0	School	24 months
Jones et al. 2015 [36]	Australia	37	male 20; female 17	9.7 ± 0.8	School	7 months
Kalarchian et al. 2009 [37]	USA	192	83 male; 109 female	10.2 ± 1.2	Medical Center	18 months
Kovacs et al. 2009 [38]	Hungary	51	23 male; 28 female	9.9 ± 1.3	School	15 weeks
Larsen et al. 2016 [39]	Denmark	115	51 male; 64 female	12.0 ± 0.4	Day camp	1 year
Li et al. 2010 [40]	China	4700	2242 female; 2458 male	9.3 ± 0.7	School	2 years
Lison et al. 2012 [26]	Spain	110	not reported	11.9 ± 2.2	Hospital	6 months
Maddison et al. 2011 [41]	New Zealand	322	male 235; female 87	11.6 ± 1.1	School	6 months
Maddison et al. 2012 [42]	New Zealand	322	male 235; female 87	11.6 ± 1.1	School	24 weeks
Maddison et al. 2014 [43]	New Zealand	251	male 142; 109 female	11.2	School/Community center	24 weeks
Magnusson et al. 2012 [44]	Iceland	321	173 male; female 148	7.3 ± 0.3	School	2 year
Monteiro et al. 2015 [45]	Brazil	32	16 female; 18 male	11.0 ± 1.5	Family based/Community	20 weeks
Nemet et al. 2008 [46]	Israel	22	14 females; 8 males	10.2 ± 0.5	Health center and nutritional clinic	3 months
Nemet et al. 2011 [47]	Israel	795	437 male; 358 female	5.2 ± 0.3	Kindergartens	12 weeks
Nowicka et al. 2009 [48]	Sweden	76	40 male; 36 female	10.5	Obesity Clinic	6 months
Safdie et al. 2013 [49]	Mexico	830	415 male; 415 female	9.7 ± 0.7	School	18 months
Simon et al. 2008 [51]	France	954	527 males; 527 female	11.7 ± 0.7	School	4 years
Sighn et al. 2009 [50]	Netherlands	1108	549 male, 559 female	12.7	School	20 month
Waters et al. 2018 [52]	Australia	2965	Not reported	8.5	School	3.5 years
Williamson et al. 2012 [53]	USA	2060	male 854; Female 1206	10.5 ± 1.2	School	28 months
Yackobovitch Gavan et al. 2009 [54]	Israel	162	81 male; 81 female	8.3 ± 1.6	Medical Center	12 weeks
Yin et al. 2012 [55]	USA	574	270 male; 304 female	8.7 ± 0.5	School	3 years
Casazza et al. 2012 [56]	USA	26	26 female	12.4 ± 0.3	Family based	16 weeks
Copeland et al. 2013 [57]	USA	699	Not reported	13.5	Not reported	24 months
Greening et al. 2011 [24]	USA	450	234 male; 216 female	8.34 ± 1.3	School	9 months
Gutin et al. 2008 [58]	USA	206	97 male; 109 female	8.5 ± −0.6	School	3 year
Johnston et al. 2009 [59]	USA	60	33 male; 27 female	12.3 ± 0.7	School	2 years
Kalavainen et al. 2011 [25]	Finland	70	28 male; 42 female	8.1 ± 0.8	Family oriented	6 months
Khan et al. 2014 [60]	USA	220	117 male; 103 female	8.8 ± 0.5	School	9 month
Lau et al. 2014 [61]	Hong Kong	48	36 male; 12 female	10.4 ± 0.9	School	6 weeks
Meyer et al. 2014 [62]	Switzerland	289	153 male; 136 female	10.6 ± 0.3	School	3 year
Marild et al. 2012 [63]	Sweden	66	28 male; 38 female	10.8 ± 1.1	School	12 month
Rush et al. 2011 [64]	New Zealand	1348	686 male; 662 female	7.5	School	2 years
Velazquez Lopez et al. 2014 [65]	Mexico	49	23 male; 26 female	11.2 ± 2.7	Family medicine Unit	16 weeks

### Effectiveness in body composition parameters

Table 2 shows the effective parameters where some degree of improvement was reported. The analysis shows 26/41 (63%) studies reported to be effective; From those 26 effective articles, 3/26 (11%) included PE in their interventions, meaning that, at least, 1 of the PE subcomponents has been used and reported as part of the PE implementation. The most repeated studied outcomes when reporting effectiveness were BMI and zBMI alone (12/26) or combined to one another or with other body composition parameters (9/26). That is 81% of the total effective articles.

**Table 2:** Type of intervention applied, studied outcome and effectiveness

	Intervention applied							Studied Outcome	Effective	Effective Outcome
	PA,D and BS	PA and BS	PA and D	D and BS	D	PA	BS			
Croker et al. 2012 [28]	√							BMI, WC, %BF	X	
Danielsen et al. 2011 [29]	√							BMI	√	BMI
Davis et al. 2012 [30]						√		zBMI, %BF, BG, IL	√	zBMI, %BF, BG, IL
Eather et al. 2013 [31]						√		zBMI, BMI	√	zBMI, BMI
Elder et al. 2013 [32]	√							zBMI,BMI,WC,%BF	X	
Foley et al. 2016 [33]							√	zBMI,BMI	X	
Gerards et al. 2015 [34]	√							zBMI,WC	X	
Hollis et al. 2016 [35]		√						zBMI,BMI	√	zBMI,BMI
Jones et al. 2015 [36]	√							zBMI,BMI,WC,%BF	√	zBMI,BMI,WC,%BF
Kalarchian et al. 2009 [37]				√				BMI,WC	√	BMI, WC
Kovacs et al. 2009 [38]						√		BMI,WC,BP	√	BMI,WC,BP
Larsen et al. 2016 [39]	√							zBMI,BMI,WC,BG	√	zBMI,BMI,WC BG
Li et al. 2010 [40]						√		zBMI,BMI,%BF	√	zBMI,BMI,%BF
Lison et al. 2012 [26]			√					zBMI,BMI,WC,%BF	√	zBMI,BMI, WC, %BF
Maddison et al. 2011 [41]						√		zBMI,BMI,WC,%BF	√	zBMI,BMI,WC,%BF
Maddison et al. 2012 [42]						√		BMI,%BF	√	BMI,%BF
Maddison et al. 2014 [43]		√						zBMI,BMI,WC,%BF	X	
Magnusson et al. 2012 [44]			√					BMI,WC,%BF	X	
Monteiro et al. 2015 [45]						√		%BF,FFM,%AF	√	%BF,FFM;%AF
Nemet et al. 2008 [46]	√							BMI,%BF	√	BMI, %BF
Nemet et al. 2011 [47]	√							BMI	X	
Nowicka et al. 2009 [48]			√					zBMI,BMI	X	
Safdie et al. 2013 [49]	√							BMI	√	BMI
Simon et al. 2008 [51]		√						BMI	√	BMI
Sighn et al. 2009 [50]	√							BMI,WC	X	
Waters et al. 2018 [52]	√							zBMI,BMI	X	
Williamson et al. 2012 [53]	√							zBMI,%BF	√	%BF
Yackobovitch Gavan et al. 2009 [54]			√					BMI,%BF	√	BMI,%BF
Yin et al. 2012 [55]						√		%BF	√	%BF
Casazza et al. 2012 [56]					√			zBMI,BMI,%BF,BG	X	
Copeland et al. 2013 [57]							√	BMI,WC,%BF	X	
Greening et al. 2011 [24]	√							BMI,WC,%BF	√	BMI,%BF
Gutin et al. 2008 [58]						√		%BF	√	%BF
Johnston et al. 2009 [59]	√							zBMI,BMI	√	zBMI,BMI
Kalavainen et al. 2011 [25]	√							BMI, WC	√	BMI,WC
Khan et al. 2014 [60]						√		zBMI,BMI,%BF	√	zBMI,BMI,%BF
Lau et al. 2014 [61]						√		BMI	X	
Meyer et al. 2014 [62]						√		%BF	X	
Marild et al. 2012 [63]	√							BMI	√	BMI
Rush et al. 2011 [64]			√					BMI,%BF,BP	X	
Velazquez Lopez et al. 2014 [65]					√			BMI,%BF,BG	√	BMI,%BF,BG

*BMI body mass index, *BP Blood pressure, *BG Blood glucose, *FFM fat-free mass, *IL insulin levels, *WC waist circumference *%BF percentage of body fat, *%AF percentage of android fat

### zBMI and BMI meta-analysis and sub-groups analysis considering PE

**Fig. 2:**
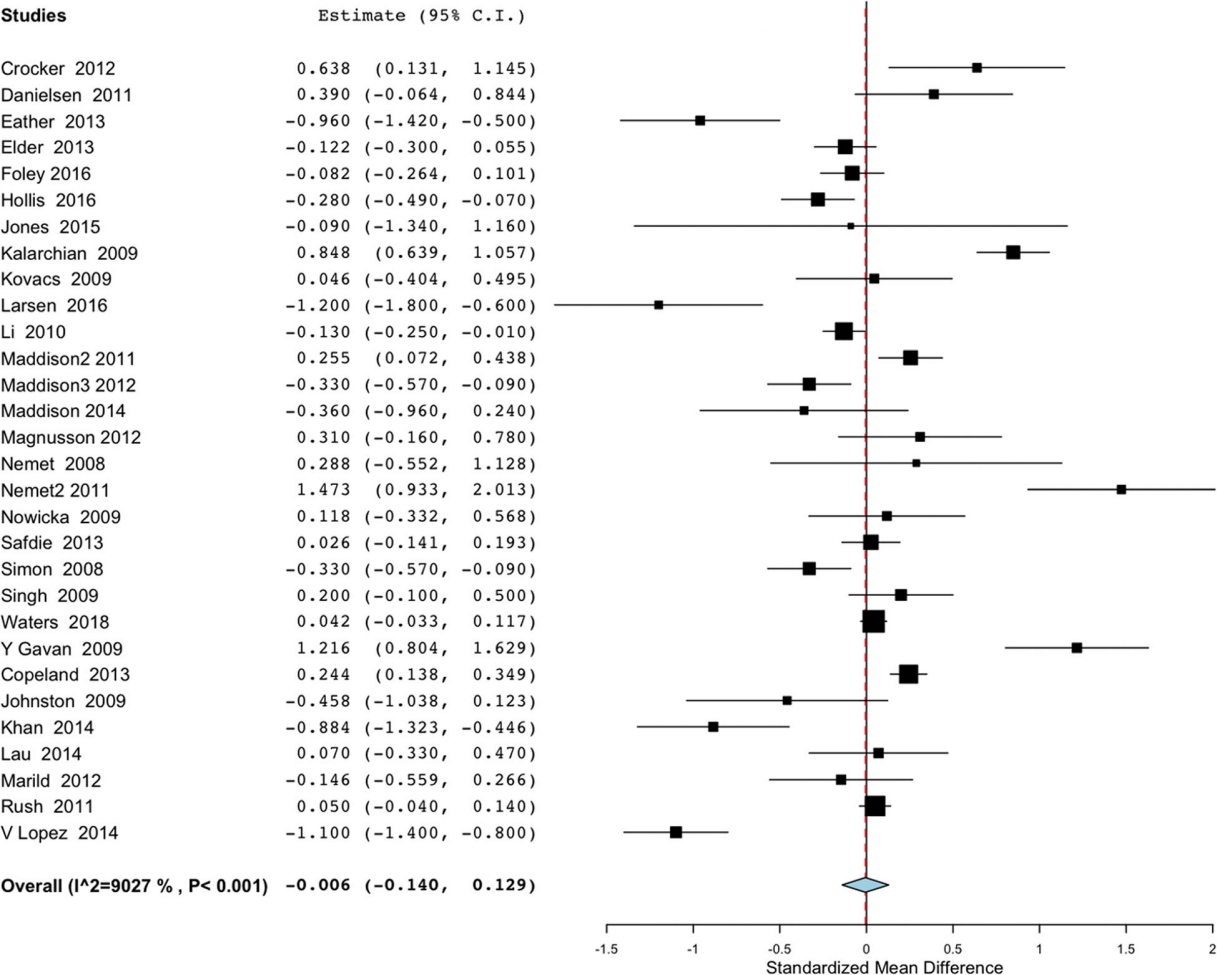
Forest plot of the overall studies using BMI

**Fig. 3:**
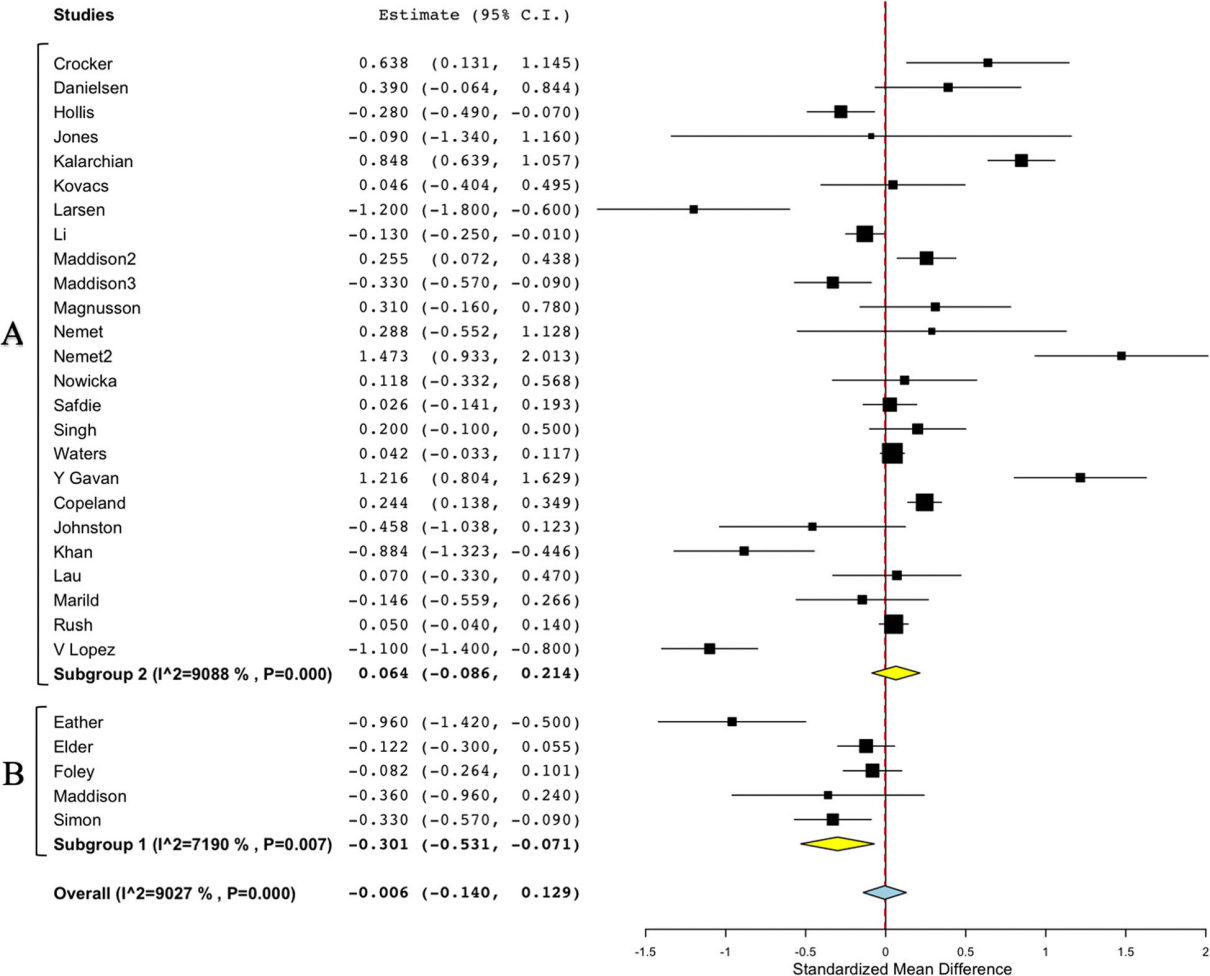
Forest plot of process evaluation on studies using BMI. Subgroup A No process evaluation; Subgroup B Process evaluation

**Fig. 4:**
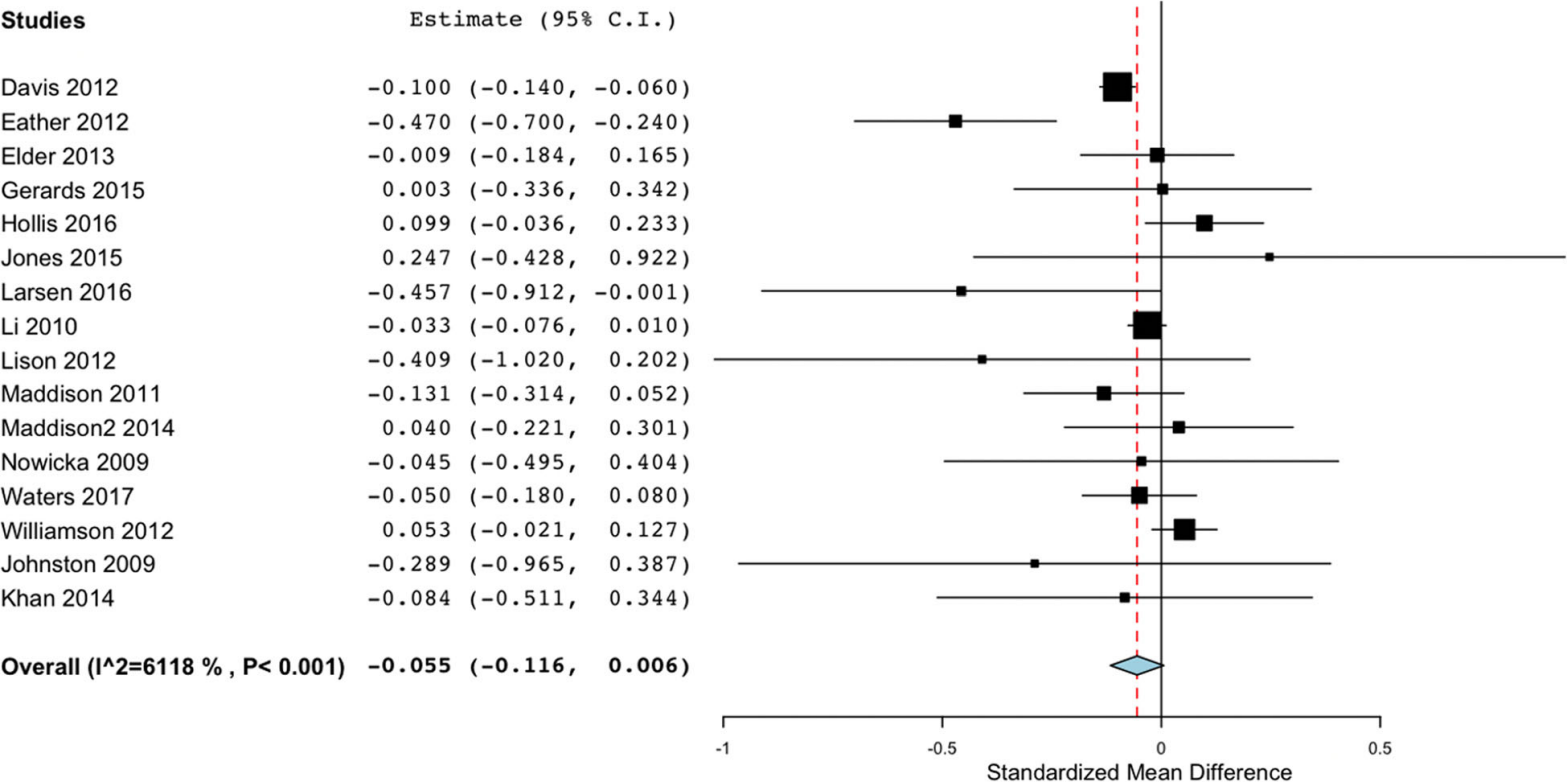
Forest plot of the overall studies using zBMI

**Fig. 5:**
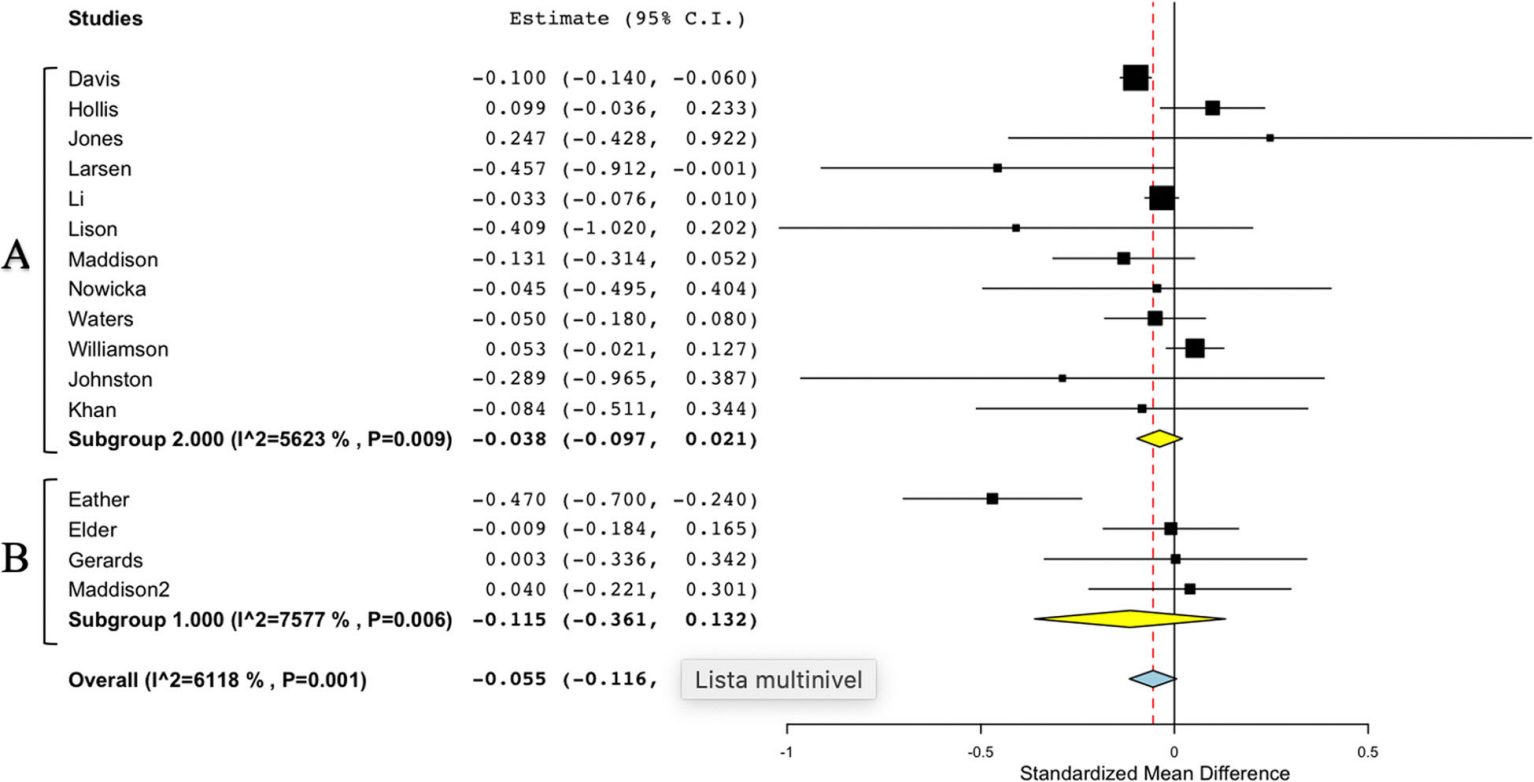
Forest plot of process evaluation on studies using zBMI. Subgroup A No process evaluation; Subgroup B Process evaluation

Figure 2 shows the overall study results and plot the global effect of changes in BMI. Figure 3 shows the sub-group studies according to the performance of PE. Figures 4 and 5 show the same overall and sub-group analysis, in this case, with zBMI. Meta-analysis of the 30 studies which reported changes from baseline to follow up in BMI found non-significant effects between control and intervention groups (Overall mean difference in BMI: − 0.055; 95% CI, − 0.116 to 0.006). The results maintained the very high heterogeneity in BMI studies (I2 = 90.27%, *p* < 0.001). Sub-group analysis of zBMI results showed significance when comparing studies including PE (− 0.301 (− 0.531, − 0.071)) versus no PE (0.064 (− 0.086, 0.214)). Heterogeneity within studies was very high (I2=. 90.27%; *p* < 0.001). Meta-analysis of the 16 studies which reported changes from baseline to follow up in zBMI found non-significant effect between studies (Overall mean difference in zBMI: − 0.055; 95% CI, − 0.116 to 0.006). Heterogeneity among studies was high (I2 = 61.18%, *p* < 0.001). The sub- group analysis results revealed non-significant differences in PE (No PE: − 0.038 (− 0.097, 0.021); PE -0.115 (− 0.361, − 0.132). In sub-group analysis, the results maintained the high heterogeneity in the PE studies (sub-group No PE: I2 = 56.23%, *p* = 0.009; sub-group PE: I2 = 75.77%, *p* = 0.006).

### Process evaluation inclusion and indicators description

In the present review all indicators have been examined and displayed in Table 3. PE was included in 17% of the studies (7/41). Fidelity and satisfaction in 4/7 (57%) studies were the indicators considered the most, followed by dose 3/7 (43%), reach 2/7 (29%) and recruitment 1/7 (13%) respectively.

**Table 3:** Process evaluation indicators use

	Recruitment Random sequence generation (Selection Bias)	Reach	Dose	Fidelity	Satisfaction
Eather et al. 2013 [31]	√	√	√	x	√
Elder et al. 2013 [32]	x	x	x	√	x
Foley et al. 2016 [33]	x	x	√	x	√
Gerards et al. 2015 [34]	x	√	√	x	√
Maddison et al. 2014 [43]	x	x	x	√	√
Simon et al. 2008 [51]	x	x	x	√	x
Yin et al. 2012 [55]	x	x	x	√	x

We also analysed how the PE indicators were presented in each paper. Eather et al. [31] reported PE in a separate paragraph within the methods section and focused on intervention workers and parents recruitment, retention, adherence and satisfaction by completing evaluation questionnaires by teachers and students. Recruitment and retention were again evaluated separately with no significant differences between study groups. Elder et al. [32] described PE in their methodology, examining process data associated to intervention fidelity with different fidelity measures for each family: “tabulations of the number and types of contacts completed” and describes it in a table with no further mention. Foley et al. [33] considered PE throughout the article and takes on a comprehensive analysis of a general implementation of PE. They focused on PE intervention at 3 levels (“from investigative team to community worker, from community worker to primary caregiver and from primary care giver to child”). Dose and satisfaction were assessed by interview with the community workers which conclusions throw that it was a “poor uptake of intervention components, and weak efficacy of the intervention itself”. Gerards et al. [34] framed PE in their results. First, they measured parental attendance in the group and telephone sessions and added PE questions in the 4-month questionnaire. Then, the participating parents completed a satisfaction questionnaire. The reports showed a “high reach as majority of lessons which were planned actually took place and the parents who did visit at least one group session, 81% (parents of 25 children) were present at 5 or more sessions” and parents had a good impression of the program and rated it on 7.7 on a 10-point scale. Maddison et al. [43] reported fidelity separately to PE in the methods section. Fidelity was assessed by monitoring the sessions performed by a researcher from the community workers with feedback to ensure all components were delivered. Primary caregivers also completed a survey to determine their perceptions of the intervention. It all showed moderate fidelity as “43% of the caregivers reported using any of the strategies to modify screen use sometimes to often”. Simon et al. [51] referred to have reported PE in a separate paper [66] in which PE is briefly described. Yin et al. [55] also referred the use of PE and explained it. They used fidelity, “feedback from the instructors on issues related to FitKit program delivery” and invites the readers to acquire the intervention manual for further research.

### Intervention programs and delivery

The intervention programs were carried out to assess 3 targets: diet, PA and BS. These 3 components were implemented either combined or alone in the intervention group during a period of time ranging from 6 weeks to 3 and a half years. Most of the programs included PA in different intensities: mild, moderate and intense, being the moderate activity the most used. The aerobic exercises included warming up, running tests, ball games, stretching etc. Few other studies also used video games or dancing lessons. The studies recorded the PA levels, in some cases, with accelerometers or pedometers and parents questionnaires. Different scales were used, such us *the* SOFIT (System for observing Fitness instruction time) [67], to measure the participants´ performance.

Diet intervention was applied predominately by holding meeting sessions to the parents of the participating children and educational workshops. Few other studies reduced calories in the school canteen through a diet plan or supervision. Collection of data using validated questionnaires and scales of food consumption such as the Food frequency questionnaire [34] were the most common used.

All BS interventions used in focus groups to obtain perceptions regarding the importance of PA and diet for children and potential barriers. Some of the targets were to reduce screen time, control of TV/computer use and motivate child’s social habits by applying positive reinforcement, environmental stimulus control and problem solving. The sessions were offered in schools and community. All the information was collected and measured in scales such us” *Psychological control scale” from Dutch version* [34], *Self-esteem scales for children* [29] *or Pierre Harris scale* [68].

### Effectiveness and use of intervention programs

From the 41 papers analysed, 16 presented a combined 3-arm target. Regarding 2-arm target, different combinations were observed: 5 studies used PA and diet, 3 studies PA and BS and 1 study in diet and BS respectively. Finally, 12 articles used PA alone, 2 diet alone and 2 BS alone. According to the results observed, more than half of interventions implementing 3 targets are effective (62%). However, PA alones showed the highest rate of effectiveness (83%). The classification according to the effectiveness in each type of target is as follows: diet, PA and BS:10/16 (62%); 2-arm target of combinations between diet, PA and BS: 5/9 (56%) and PA alone 10/12 (83%) respectively. Finally, in less proportion, effectiveness was shown in diet alone 1/2 and BS alone in 0/2 articles.

### Quality assessment risk of Bias

**Fig. 6:**
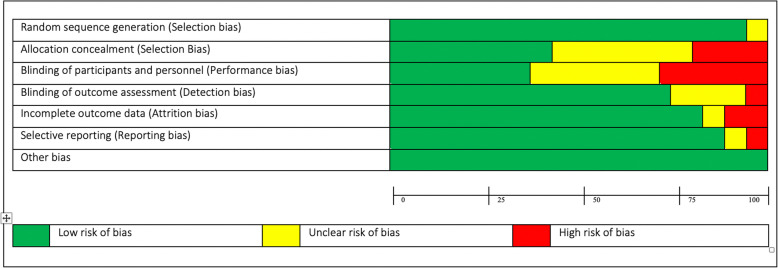
Risk of bias graph

**Fig. 7:**
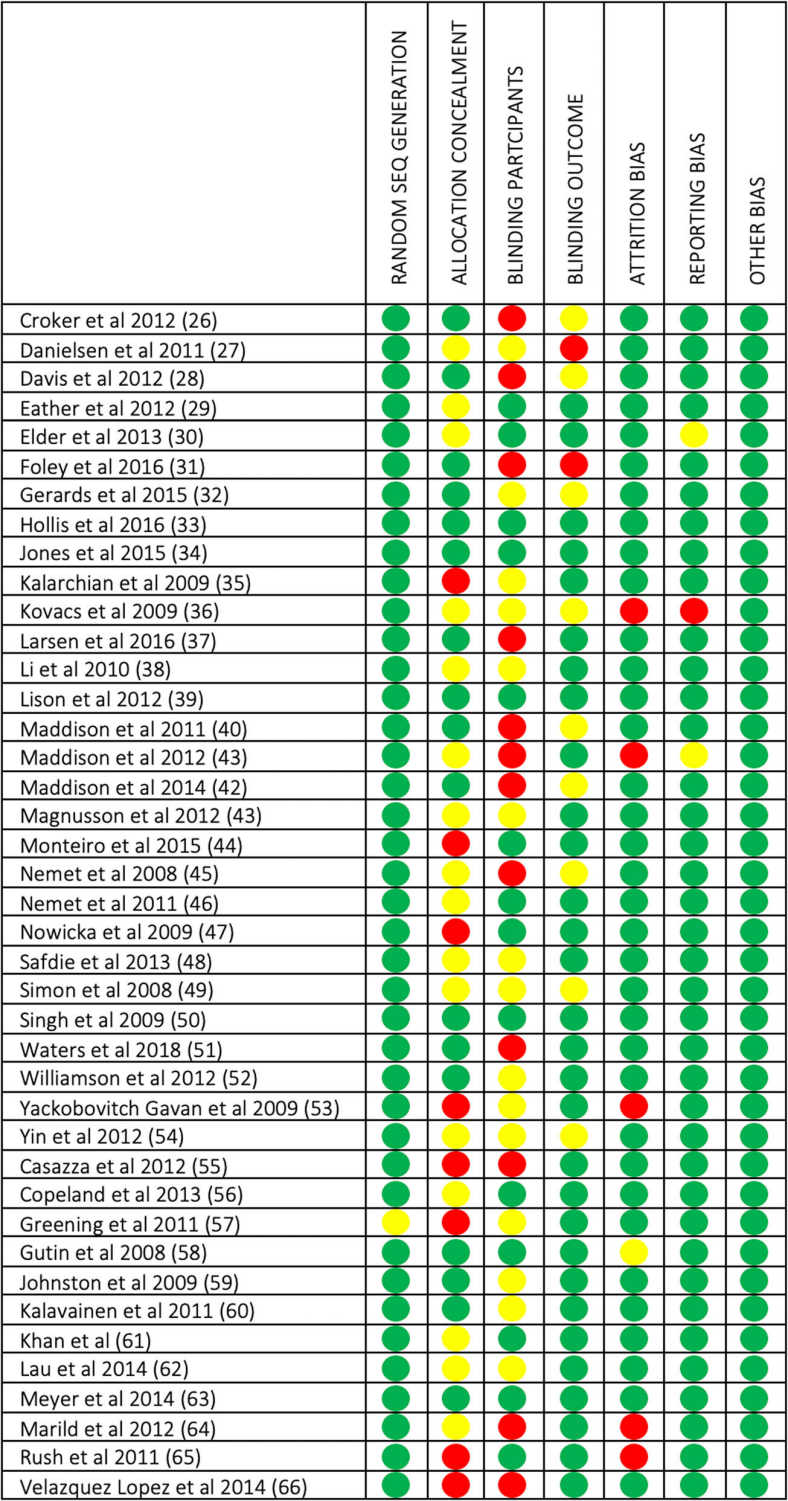
Summary risk of bias

Figures 6 and 7 present a risk of bias summary within included studies. 17/41 (41%) of studies scored high risk (3-arm intervention 7/16 (44%); 2-arm intervention 5/9 (56%) and PA alone 5/12 (42%). Only 5/41 (13%) studies reported low risk (3-arm intervention 2/16 (12%); 2-arm intervention 2/9 (22%) and PA alone 1/12 (8%). Concerning random sequence generation, all types of intervention used had no differences (low risk, 3-arm intervention: 15/16 (94%); 2-arm intervention: 9/9 (100%) and PA alone: 12/12 (100%). Concerning the allocation concealment (low risk, 3-arm intervention: 9/16 (56%), 2-arm intervention: 4/9 (44%) and PA alone 4/12 (33%). On the other hand, effective studies scored almost half high risk 20/41 (49%), from which 13/26 (50%) were effective and 7/15 (47%) non-effective. Moreover, 16/41 (39%) studies reported to have unclear risk: effective 10/26 (38%); non-effective 6/15 (40%). Only 5/41 (13%) studies reported to have low risk: effective 3/26 (11%); non-effective 2/15 (13%). Regarding random sequence generation, there was no difference according to the quality of the studies. Effective: low risk 25/26 (96%), unclear risk 1/26 (4%) and high risk 0/26 (0%); non-effective: low risk 15/15 (100%), unclear risk 0/15 (0%) and high risk 0/15 (0%). Concerning the allocation concealment domain, there were no differences found either. Effective: low risk 11/26 (42%), unclear 10/26 (38%) and high risk 5/26 (19%); non-effective: low risk 7/15 (47%), unclear 5/15 (33%) and high risk 3/15 (20%).

## Discussion

### Principal findings

Meta-analysis showed that, overall, recent studies preventing obesity and T2DM are not effective in terms of BMI and zBMI. After sub-group analysis, those studies reporting PE showed positive changes in terms of BMI and those not reporting PE did not show changes in terms of BMI and zBMI. Moreover, fidelity and satisfaction were the 2 PE indicators identified which were most implemented in those articles considering PE. Lastly, the 3-arm target interventions were the most used while the interventions implementing PA alone were the most effective of all.

Interventions aiming to prevent childhood obesity use different outcome variables. The most widely used are those based on anthropometric measurements. The most used anthropometric index is the BMI. However, it has important limitations as it does not distinguish between fat mass and lean mass [69, 70]. Despite this limitation, Cole et al. showed that BMI could be the best parameter for measuring changes in adiposity [71]. As the majority of the included studies used BMI and zBMI as the main outcome variable, meta-analysis was only performed for BMI and zBMI.

In the report of effectiveness, PE should be included in order to allow comparability with other prevention studies. To date, few studies have shown a comprehensive evaluation on how the interventions are implemented or provided a full report of the findings after the PE was carried out [72]. PE is necessary to validate the implementation program structure in order to interpret the final outcome. In the present review, feedback of the PE implementation outcome is generally incomplete and briefly discussed. It has also been observed in the present review that there is high heterogeneity of PE reporting. Despite a comprehensive analysis of the reported PE findings it is challenging to obtain practical information in order to improve future intervention studies. In order to work on the same line of action, ideal PE reporting should provide a comprehensive evaluation in both the study protocol and other related articles. Thus, it would be easy to identify how the PE indicators have been applied and how the overall evaluation has been performed. Lloyd et al. published in a separate paper a PE assessment of a study aiming to prevent childhood obesity [15]. This article concluded that using a structured pathway to report PE in every complex intervention could lead to successfully scale up the same guidance to other school-based interventions in other community studies and perform the intervention as designed.

When evaluating multi-component interventions, a systematic review of Brown et al. [73] showed that multi-target interventions focused on changing dietary and PA patterns in children had the highest proportion of effective studies. In Mead et al. [74] systematic review, it was observed that “multi-component behavior-changing interventions that incorporate diet, PA and behavior change might be beneficial in achieving small, short-term reductions in BMI, zBMI and weight in children aged 6 to 11 years old”. Moreover, Frübeck et al. stated that an intervention implemented at 3 different levels of diet, PA and BS is proved to be the most effective [75]. However, it has been observed in the present systematic review that PA alone has been reported as the intervention with the most effective results. This fact might be due to the complexity to carry out an intervention at 3 different levels, lack of sources and financial support over time or lack of a continuous evaluation of the implementation performed. It might also be possible that PA recommendations in 3-arm interventions were more advise oriented and mild intensity [34] whereas PA alone intervention was predominantly focused on moderate to high intensity [60].

One of the most frequent and deleterious complications of obesity is T2DM. According to Liese AD et al. [76] “T2DM is no longer just a disorder of mature age, there is now a well-recognized trend toward younger people presenting with this disease”. The diabetes unfavorable effects on morbidity and mortality are more prominent among patients being diagnosed at a younger age comparing them with the first diagnosis of T2DM, usually at an adult stage. Therefore, taking to consideration these results, we must emphasise the increasing need to unite all efforts to develop effective interventions focusing on young to middle age population [77]. According to Manios Y et al., T2DM has a strong association to obesity and the risk of chronic diseases when sedentary behavior is established among youth [78]. For these reasons, new studies should consider interventions to prevent in the long term, both obesity and T2DM. Despite that some included studies aimed to prevent not only obesity but also T2DM, from childhood, the current length of the follow up period was not enough to assess the preventive efficacy in terms of T2DM.

Concerning the quality of the studies in this review, there was in general, a limited number of participants in experimental studies and predominantly reduced periods of follow-up. For these reasons, the majority of studies were classed as poor or moderate methodological quality and high risk of bias.

### Limitations and strengths

Although this review increases the knowledge on the relevance of the PE, it has some limitations. The present systematic review has followed the recommendations of PE use published by Saunders et al. [19] and Moore et al. [20]. However, the PE framework is currently in the process of development and several authors claim different names, criteria and indicators with no consensus reached. Although most of PE indicators share a common range of action, allocate certain data collected in the pertinent PE indicator remains a challenge. Additionally, we assume that the search strategy might not have considered all existing manuscripts including PE, as not all interventions will report PE or `process assessment´ within the manuscript. Therefore, the present search strategy relied on studies that included PE reporting in the same manuscript. Moreover, it has been observed that most of the included articles did not provide a systematic PE of the intervention. When PE was performed, it was not implemented according to the guidelines followed in this review, as most of the indicators were not considered. Therefore, all articles performing at least 1 PE indicator were considered as PE inclusion. Another limitation might be found in the low number of articles included in the initial search of large-scale topics such as obesity and T2DM. This might be due to the fact that key words related to PE were also included when applying the search terms. It should also be considered that T2DM was included with the same relevance as obesity, rather than studying obesity alone. However, the majority of articles did not perform a combined analysis of both obesity and T2DM, being the former the predominant studied outcome.

Despite Cochrane guidelines provide an exhaustive view of how to use the tool and recommends consensus between the reviewers, subjective decision making is also involved, meaning that the criteria is also subject to personal input [23]. Therefore, several risk-of-bias assessments may be needed for each study. We have not yet formulated recommendations on which results should be targeted with an assessment, or how many results should be assessed [22]. The assessment of risk of bias is specific to a particular result, for a particular outcome measured and at a particular time. This could affect authors when extracting information that implies relevance to risk of bias from study reports [22].

The present study also had some strengths. Firstly, to our knowledge, this is the most comprehensive and up-to-date overview of children obesity and T2DM prevention programs, considering in some cases, the inclusion of PE assessment. The target of this study was to show the important role of PE in order to avoid mislead information. Secondly, the review included exclusively RCTs, regarded as the best design in complex interventions. Lastly, this study evaluated a combination of obesity and T2DM risk in children, attempting to tackle the 2 most important diseases affecting the present and future of sedentary children and adolescents [79].

## Conclusions

Overall, obesity and T2DM prevention studies included in this review are not effective in terms of BMI and zBMI. Those studies performing PE reported to be effective in terms of BMI, while studies not reporting PE did not have positive results in terms of BMI and zBMI. In addition, there was a low degree of PE implementation as none of the intervention studies included all PE indicators, and those studies including PE in their interventions, did not provide full report of the PE subcomponents. Further research is needed in order to promote PE inclusion in all health intervention programs and to provide a more robust evaluation of the program implementation and effectiveness.

## Supplementary Information

**Additional file 1.** PRISMA checklist

## Data Availability

Not applicable.

## Abbreviations

%BF: Percentage body fat
%AF: Percentage android fat
BG: Blood glucose
BMI: Body mass index
BP: Blood pressure
BS: Behavioral support
D: Diet
EBRBs: Energy balance-related behaviours
FFM: Fat-free mass
PA: Physical activity
PE: Process evaluation
RCT: Randomized control trial
T2DM: Type 2 diabetes Mellitus
WC: Waist circumference
